# Supplementary material for: CryoEM structure of the super-constricted two-start dynamin 1 filament
Source: Nat Commun. 2021 Sep 13;12:5393. doi: 10.1038/s41467-021-25741-x (PMC8437954; doi:10.1038/s41467-021-25741-x)
Supplement: Supplementary file 1 — Supplementary information [file 41467_2021_25741_MOESM1_ESM.pdf]

Supplementary Table 1 | Summary of representative mutant data from literatures.

locations	mutants	Impacts			Reference
		GTPase†	Filament Assembly‡	Transferrin uptake#	
Stalk interface 1	L330R/Q334R/L702R			significantly lowered	1
Stalk Interface 2	I481D/H687D/L688S		Deficient		2
Stalk Interface 3	F403A	no stimulation		significantly lowered	2
	D406R/M407R/T488W			significantly lowered	1
	S347A/G348A/D349A	wild type		wild type	2
	Q350A/V351A/D352A/T353A*	no stimulation		lowered	2
	L354A/E355A/L356A/S357A*	no stimulation		significantly lowered	2
	G358R	no stimulation		significantly lowered	2
	G397D	no stimulation	Deficient		3
	R399A	no stimulation	Defective		4
	IHGIR395-399AAAAA		Deficient	Significantly lowered	5

†GTPase denote *in vitro* biochemical analysis
‡ Filament Assembly denote *in vitro* biochemical analysis
# Transferrin Uptake denotes *in vivo* cellular analysis.
* L354 in dyn3, Y354 in dyn1; V351 in dyn3, I351 in dyn1

Supplementary Table 2 | List of primers used in this study.

Primer Name	Sequence (5’ to 3’)	Comment
**EcoR-Kzk-MBP**	GGTGGAATTCTGCAGATATCGCCACCATGAAAATCGAAGAAGGTAAACTG	Forward primer to generate MBP gene fragment
**H6-PP-dynamin**	TTGCCCATGGTACCGGGCCCCTGGAAC	Reverse primer to generate MBP gene fragment with overlap sequence with dynamin
**PP-dynamin**	CCGGTACCATGGGCAACCGCGGCATG	Forward primer to generate dynamin1 gene fragment
**dynamin-XbaI**	GGTTTAAACGGGCCCTCTAGATTAGACGGTGGTCGTGTTGATGTTG	Reverse primer to generate dynamin1 gene fragment

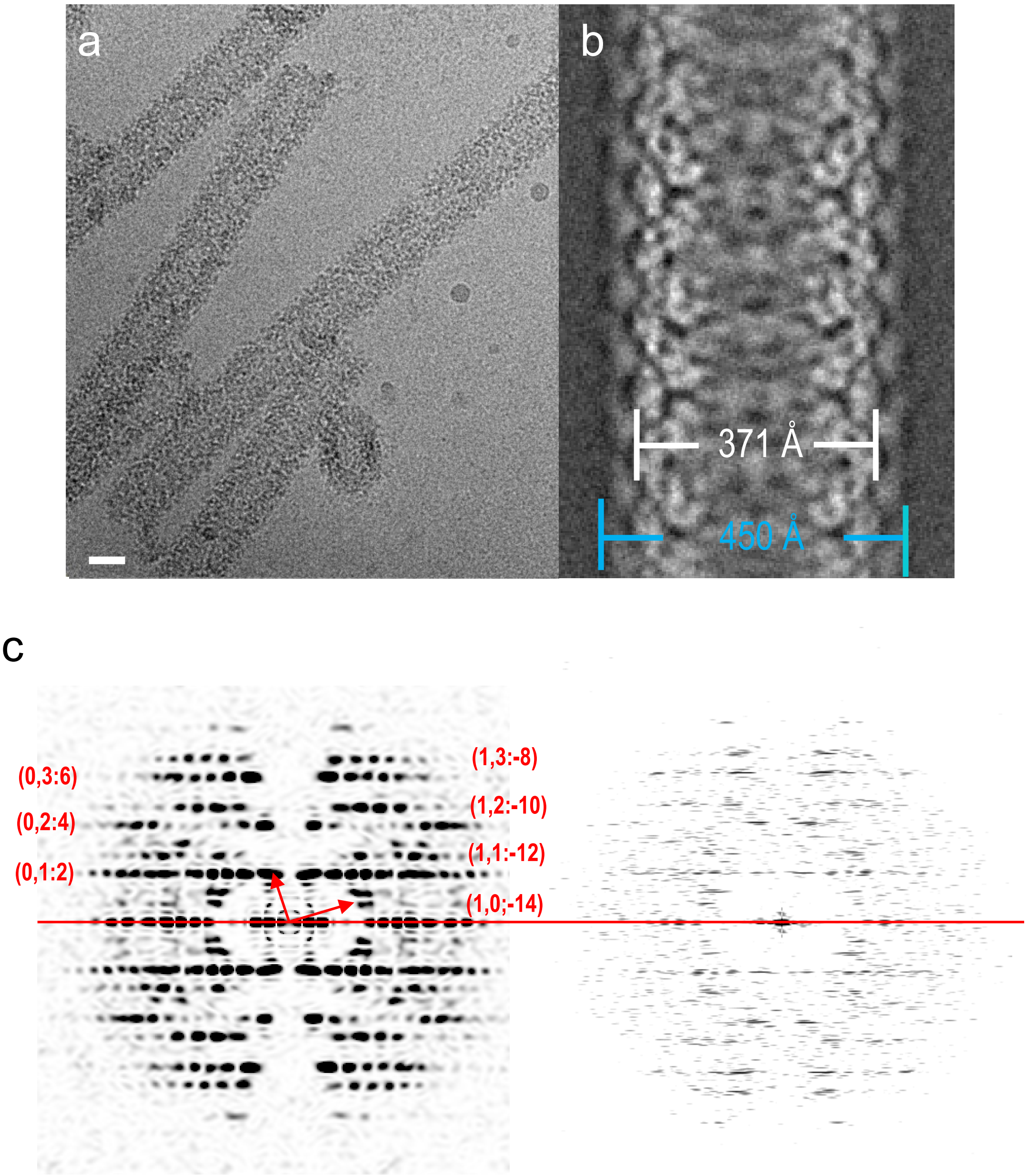

Supplementary Figure 1 | CryoEM of MBP-Dyn1ΔPRD assembly. a) A low-dose projection image of MBP-Dyn1ΔPRD tubular assembly. Scale bar, 250 Å. Three and more independent experiments were carried out. b) A 2D class average of MBP-Dyn1ΔPRD tubes with indicated outer diameters for the boundary of dynamin (white) and of the maltose-binding protein tag (blue). c) Fourier transforms of the class average in B (left) and of a raw image of a single tube (right). The tube belongs to a 2-start helical family of (-14, 2). Helical indices are shown. Red arrows point to the two vectors (1,0; -14) and (0,1;2) in helical indexing.

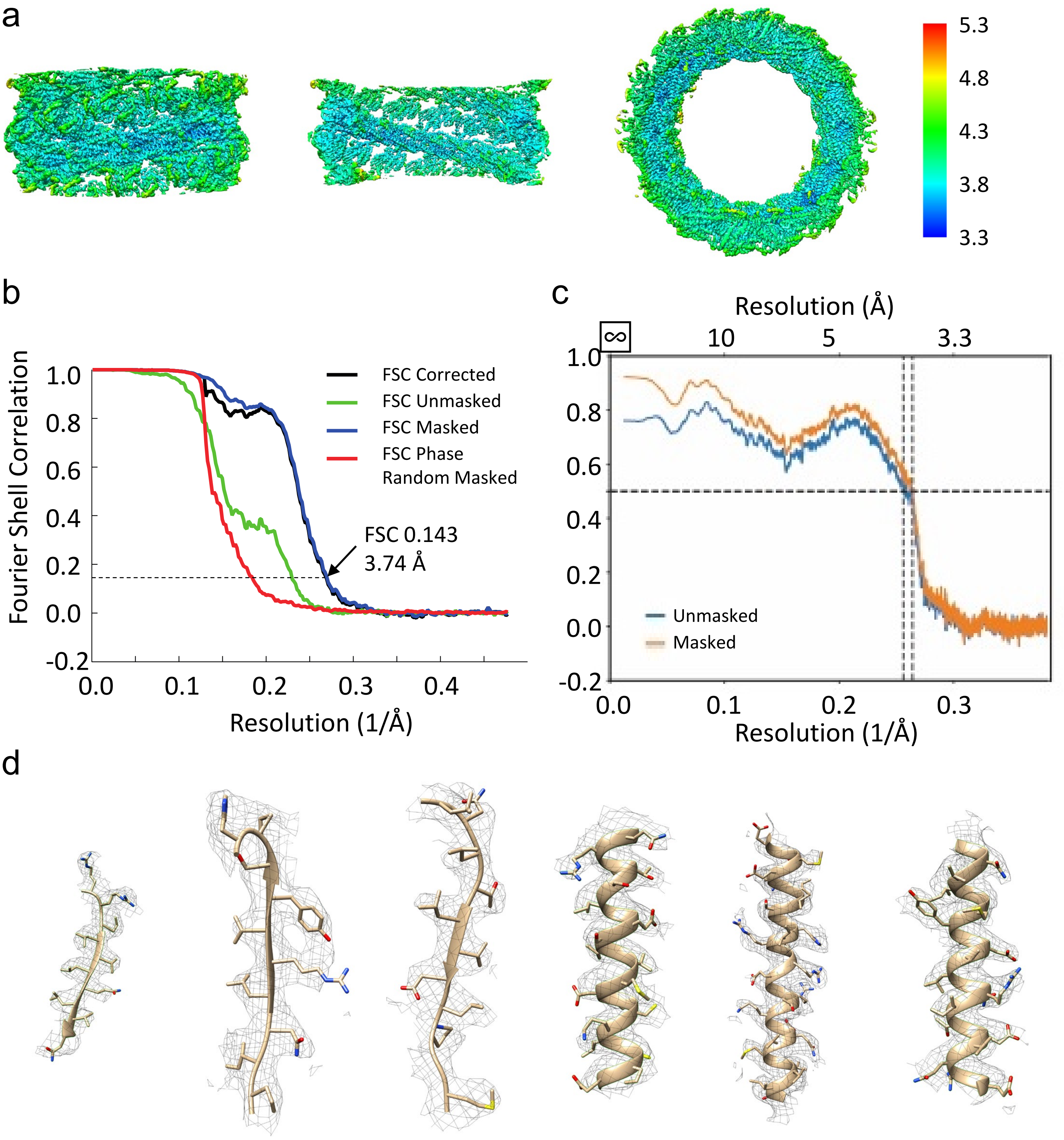

Supplementary Figure 2 | Map and model quality. a) Local resolution estimation of the cryoEM map of the MBP-Dyn1ΔPRD filament, showing the side (left), cross section (middle) and axial (right) views. Map is locally sharpened by RELION 3.0 and contoured at 3σ b) FSC curves of the density map of the MBP-Dyn1ΔPRD filament. c) Map-to-model FSC curves from Phenix. d) Representative PostProcesse sharpened density map from RELION 3.0 overlapped with the refined atomic model. The maps are contoured between 1-2σ.

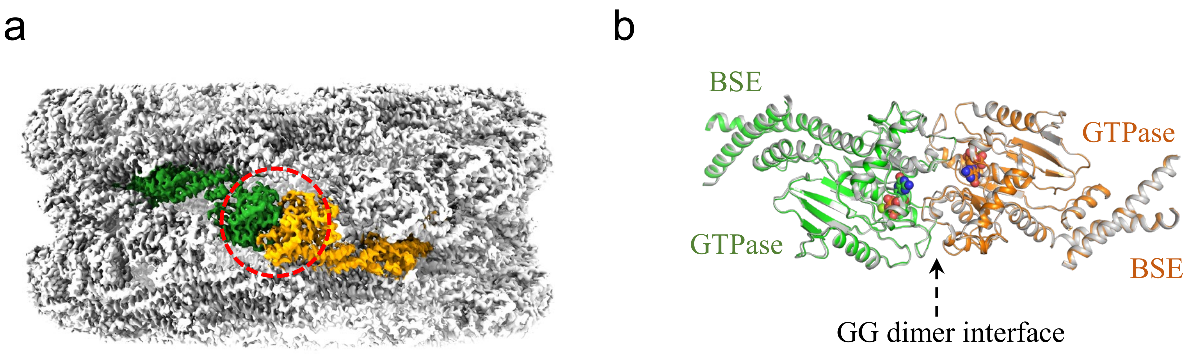

Supplementary Figure 3 | Conserved GG dimeric interfaces of the Dyn1ΔPRD helical assemblies. a) Density map of a GG dimer in two-start assembly. The two monomers are colored in green and orange. **b)** Overlap of GG dimer interfaces (circled in a) from one-start (grey) and two-start (green and orange) Dyn1ΔPRD assemblies. The nucleotides GMP-PCP are shown as spheres.

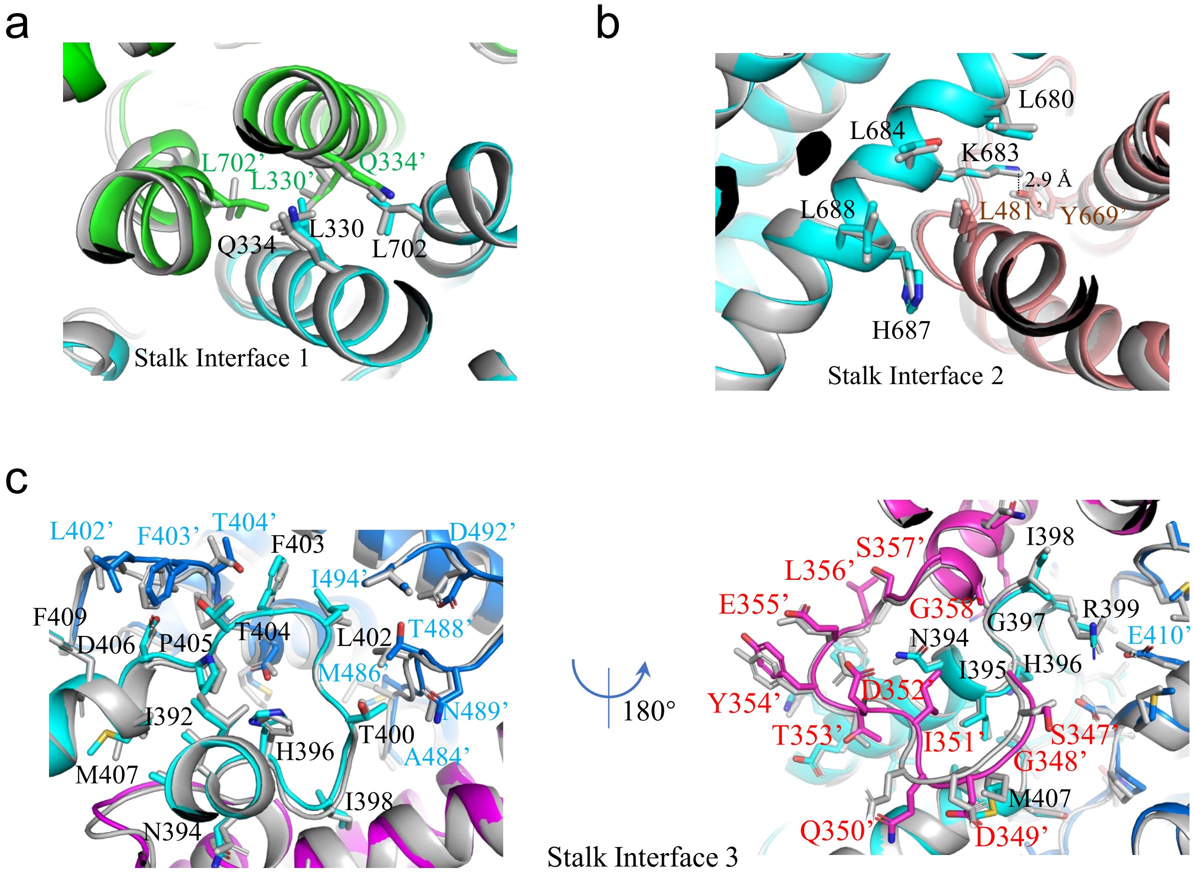

Supplementary Figure 4 | Structural and functional analysis of the three conserved Stalk interfaces. a-c) Structural comparisons of the three Stalk interfaces between the one-start and the two-start helical structures. The Stalk of the one-start helix are colored in light grey, and that of the two-start helix are colored by chain. The chain colored in cyan is aligned.

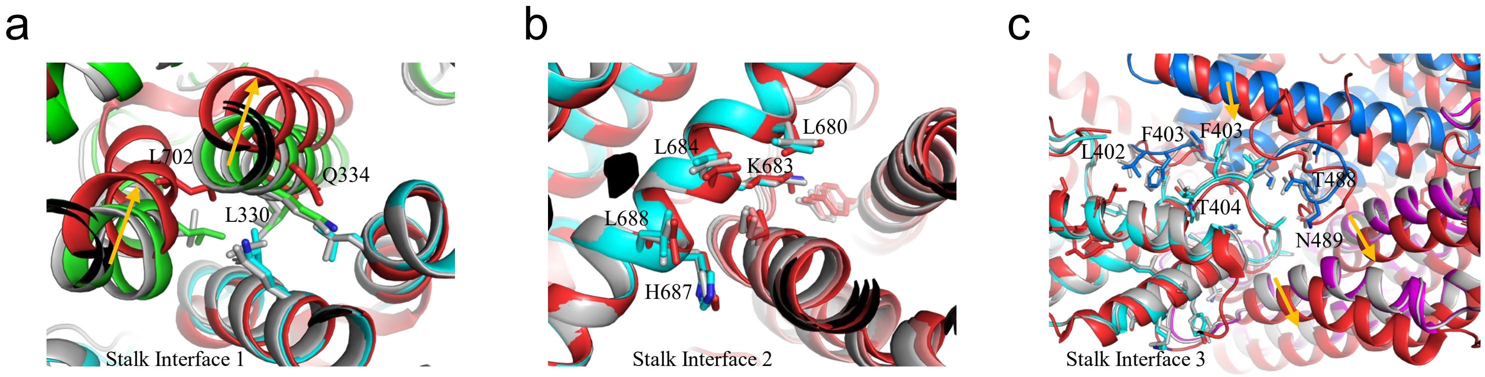

Supplementary Figure 5 | Comparisons of the Stalk interfaces between the cryo-EM helical structures and the crystallographic tetramer structure (5A3F). The Stalk of the one-start helix are colored in light grey, and that of the two-start helix are colored by chain, and that of the crystallographic tetramer is colored in red. Yellow arrows indicate the relative conformational changes. The chain colored in cyan is aligned. The views are in similar angles as in Supplementary Figure 4.

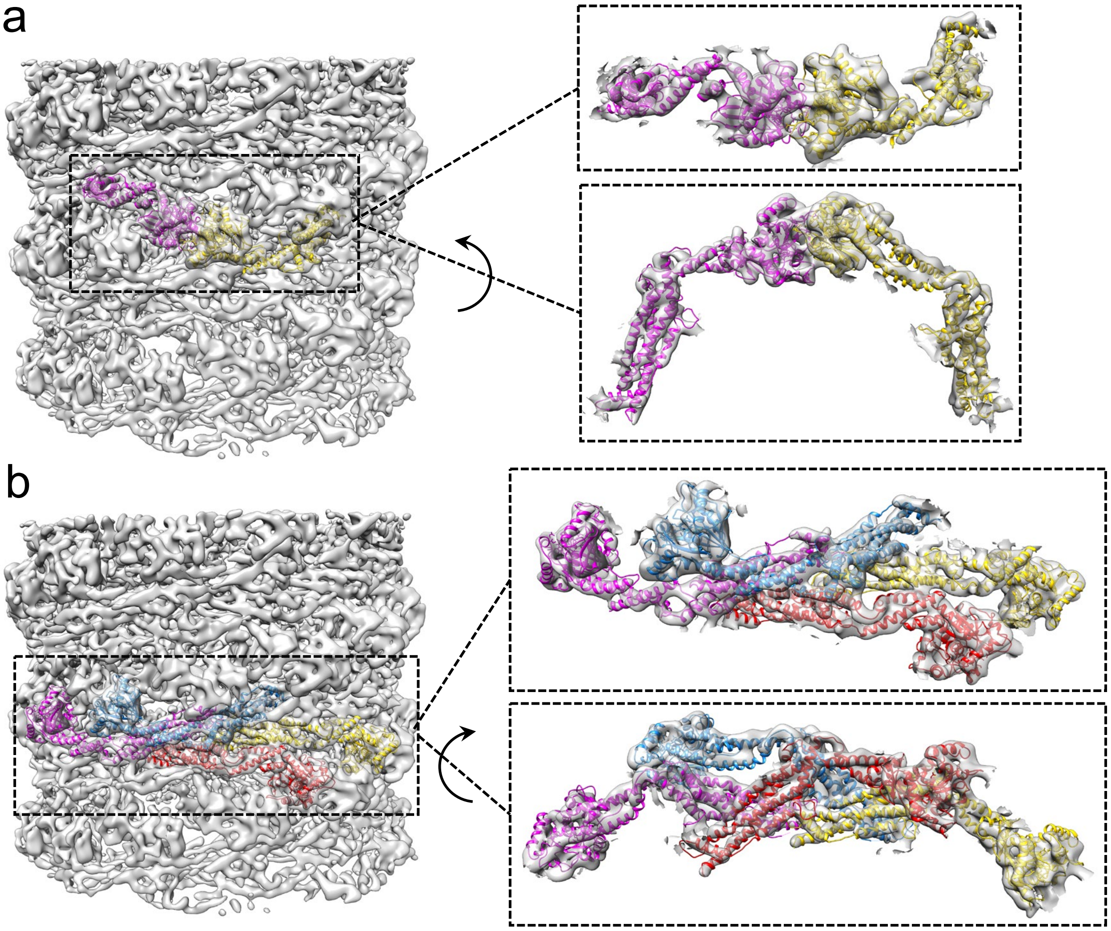

Supplementary Figure 6 | Fitting the two-start lipid-free structures into the 10.1 Å resolution two-start lipid-bound helix map (EMDB-7958). a) Fitting the GG dimer. b) Fitting the tetramer. The difference in the helical symmetry parameters (rise 14.63 Å and twist 26.14° for EMDB-7958; rise 13.58 Å and twist 24.43° for the two-start helix reported in this study) may reflect a small conformation adjustment upon lipid binding or may come from data processing. No significant difference could be observed at the tetrameric structure between the two two-start helical maps.

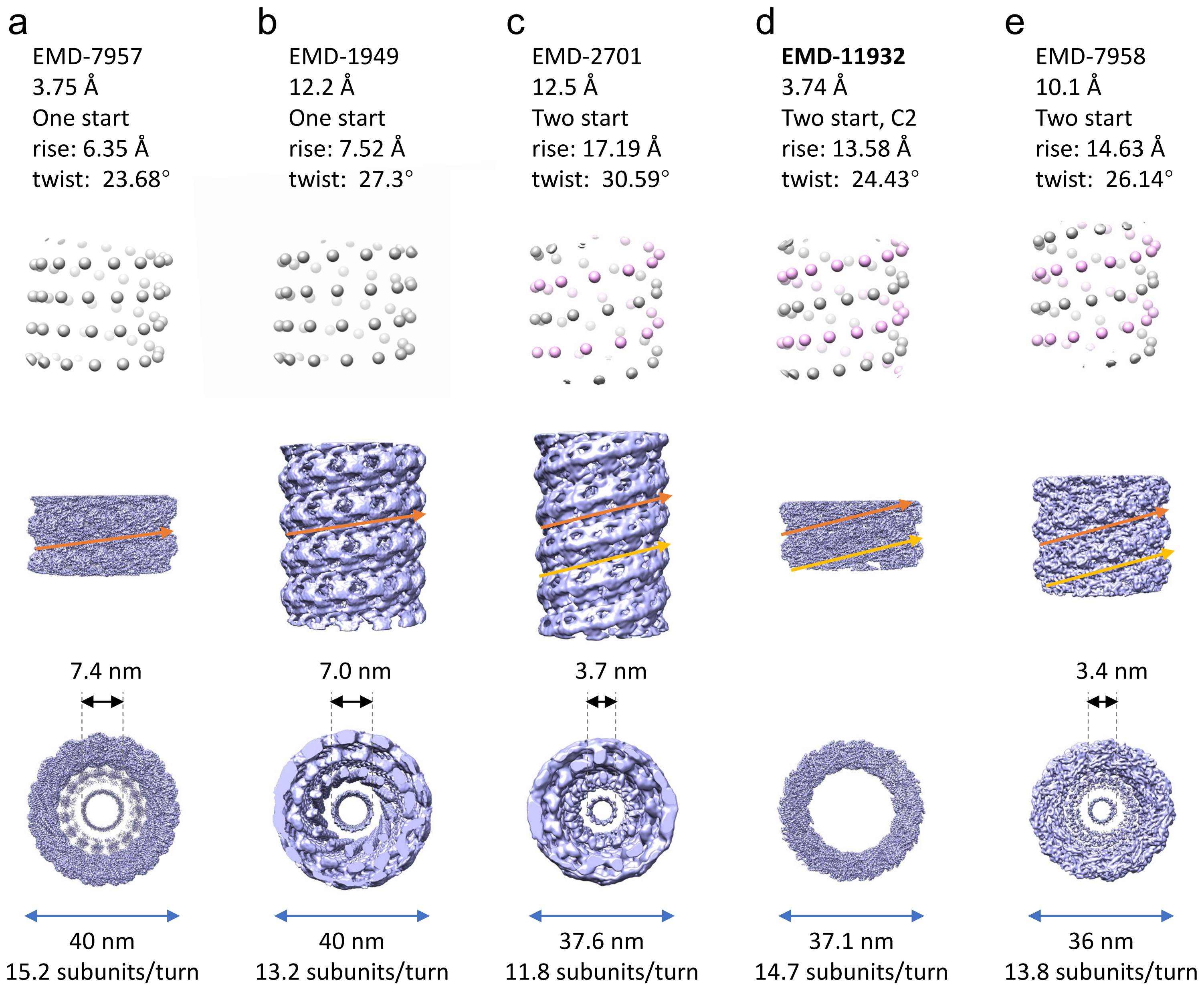

Supplementary Figure 7 | Cryo-EM reconstructions of constricted and super-constricted dynamin filaments. For each column, the EMD code, map resolution and helical symmetry parameters of each reconstruction is listed on top. The EMD code for this study is in bold. Schematic beads model of the helical symmetries are then shown. For the two-start reconstructions, the beads belongs to the two rungs are colored in grey and pink. Side and top views with outer filament and inner lipid tube diameters are shown. The comparisons of different cryo-EM reconstructions of dynamin filaments were also discussed in Ford et al., except the beads model and the new atomic two-start reconstruction in (d). a-b) Dyn1ΔPRD in complex with GMP-PCP and DOPS. c) Dyn1ΔPRD K44A in complex with GTP and DOPS. d) Dyn1ΔPRD in complex with GMP-PCP. e) Dyn1ΔPRD in complex with GTP and DOPS. Different reconstructions are arranged from the left to the right in the decrease of the outer and the inner diameters.

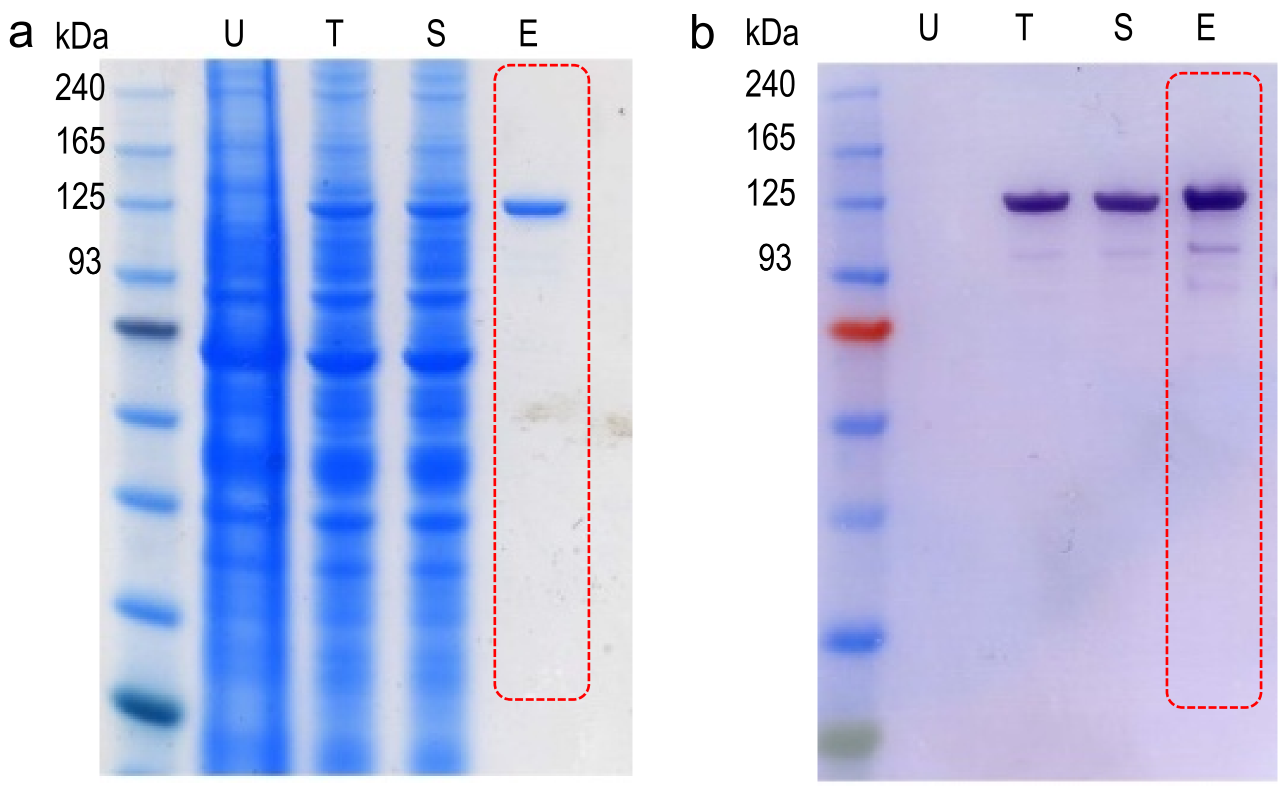

Supplementary Figure 8 | Uncropped gels and blots shown in this study, red dashed lines denote the cropped region. a) Gels shown in Figure 1c. b) Blots shown in Figure 1c. Untransfected cells (U), transfected cells (T), soluble fraction of cell lysate (S) and elution from amylose affinity resin (E). Molecular weight (MW) markers are shown in kilodaltons (kDa).

Supplementary References

1. Kong, L. *et al.* Cryo-EM of the dynamin polymer assembled on lipid membrane. *Nature* **560**, 258-262, doi:10.1038/s41586-018-0378-6 (2018).
2. Reubold, T. F. *et al.* Crystal structure of the dynamin tetramer. *Nature* **525**, 404-408, doi:10.1038/nature14880 (2015).
3. Ford, M. G., Jenni, S. & Nunnari, J. The crystal structure of dynamin. *Nature* **477**, 561-566 (2011).
4. Ramachandran, R. *et al.* The dynamin middle domain is critical for tetramerization and higher-order self-assembly. *The EMBO journal* **26**, 559-566, doi:10.1038/sj.emboj.7601491 (2007).
5. Faelber, K. *et al.* Crystal structure of nucleotide-free dynamin. *Nature* **477**, 556-560, doi:10.1038/nature10369 (2011).
