# Supplementary figures and images for: CryoEM structure of the super-constricted two-start dynamin 1 filament
Source: Nat Commun. 2021 Sep 13;12:5393. doi: 10.1038/s41467-021-25741-x (PMC8437954; doi:10.1038/s41467-021-25741-x)

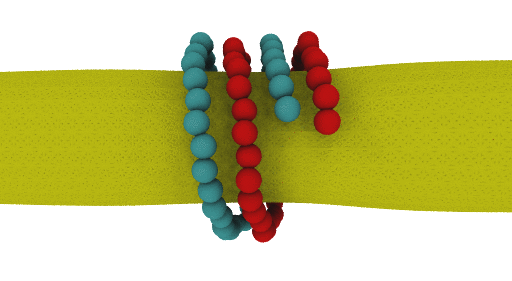

Supplement: Supplementary file 3 — Supplementary Movie 1 [file 41467_2021_25741_MOESM3_ESM.gif]
