# Supplementary material for: CryoEM structure of the super-constricted two-start dynamin 1 filament
Source: Nat Commun. 2021 Sep 13;12:5393. doi: 10.1038/s41467-021-25741-x (PMC8437954; doi:10.1038/s41467-021-25741-x)
Supplement: Supplementary file 4 — Description of additional supplementary files [file 41467_2021_25741_MOESM4_ESM.docx]

Description of additional supplementary files

Title: Supplementary Movie 1

Description: Simulation of active constriction by a twostart helix. This proof of concept simulation demonstrates that the motor torques are non-cancelling within a two-start helix and that the motor activity within a two-start helix can generate constriction. The two filaments (red and cyan), each with a pitch of 20 nm, are initially wrapped around an elastic membrane tube (yellow) at its equilibrium diameter (40 nm). Then, in the presence of 300 µM GTP, the system actively constricts to an inner diameter of 8 nm in 300 milliseconds. The consequence of the one-start like geometry that is seen at the filament terminals at the end of the simulation is an interesting subject for future research.
