# Supplementary material for: CryoEM structure of the super-constricted two-start dynamin 1 filament
Source: Nat Commun. 2021 Sep 13;12:5393. doi: 10.1038/s41467-021-25741-x (PMC8437954; doi:10.1038/s41467-021-25741-x)
Supplement: Supplementary file 5 — Reporting Summary [file 41467_2021_25741_MOESM5_ESM.pdf]

nature research

Corresponding author(s): Peijun Zhang

Last updated by author(s): Aug 19, 2021

Reporting Summary

Nature Research wishes to improve the reproducibility of the work that we publish. This form provides structure for consistency and transparency in reporting. For further information on Nature Research policies, see our Editorial Policies and the Editorial Policy Checklist.

Statistics

For all statistical analyses, confirm that the following items are present in the figure legend, table legend, main text, or Methods section.

n/a	Confirmed	
☐	☒	The exact sample size (*n*) for each experimental group/condition, given as a discrete number and unit of measurement
☐	☒	A statement on whether measurements were taken from distinct samples or whether the same sample was measured repeatedly
☒	☐	The statistical test(s) used AND whether they are one- or two-sided<br>*Only common tests should be described solely by name; describe more complex techniques in the Methods section.*
☒	☐	A description of all covariates tested
☐	☒	A description of any assumptions or corrections, such as tests of normality and adjustment for multiple comparisons
☐	☒	A full description of the statistical parameters including central tendency (e.g. means) or other basic estimates (e.g. regression coefficient) AND variation (e.g. standard deviation) or associated estimates of uncertainty (e.g. confidence intervals)
☒	☐	For null hypothesis testing, the test statistic (e.g. *F*, *t*, *r*) with confidence intervals, effect sizes, degrees of freedom and *P* value noted<br>*Give P values as exact values whenever suitable.*
☒	☐	For Bayesian analysis, information on the choice of priors and Markov chain Monte Carlo settings
☒	☐	For hierarchical and complex designs, identification of the appropriate level for tests and full reporting of outcomes
☒	☐	Estimates of effect sizes (e.g. Cohen's *d*, Pearson's *r*), indicating how they were calculated

Our web collection on statistics for biologists contains articles on many of the points above.

Software and code

Policy information about availability of computer code

Data collection	EPU (available with the instrument).
Data analysis	CryoEM data processing: Scipion, RELION3; CTFFIND4; MotionCor2 (available and referenced in methods).<br><br>Model building: Coot 0.9.4.1; Phenix 1.18.2-3874; Chimera 1.15 and ChimeraX 1.1 (available and referenced in methods).

For manuscripts utilizing custom algorithms or software that are central to the research but not yet described in published literature, software must be made available to editors and reviewers. We strongly encourage code deposition in a community repository (e.g. GitHub). See the Nature Research guidelines for submitting code & software for further information.

Data

Policy information about availability of data

All manuscripts must include a data availability statement. This statement should provide the following information, where applicable:

- Accession codes, unique identifiers, or web links for publicly available datasets
- A list of figures that have associated raw data
- A description of any restrictions on data availability

The CryoEM density map of Dyn1ΔPRD has been deposited in the EM Data Bank (EMDB) under the accession code EMD-11932 (https://www.ebi.ac.uk/emdb/entry/EMD-11932). The atomic model of the Dyn1ΔPRD helix has been deposited in the Protein Data Bank (PDB) under the accession code 7AX3 (https://www.rcsb.org/structure/7AX3).

April 2020

Field-specific reporting

Please select the one below that is the best fit for your research. If you are not sure, read the appropriate sections before making your selection.

☒ Life sciences ☐ Behavioural & social sciences ☐ Ecological, evolutionary & environmental sciences

For a reference copy of the document with all sections, see nature.com/documents/nr-reporting-summary-flat.pdf

Life sciences study design

All studies must disclose on these points even when the disclosure is negative.

Sample size	For cryoEM structure determination, sample sizes were those required for the resolution. The details of datasets, including sample sizes, are listed in table 1.
Data exclusions	For cryoEM 3D reconstruction, standard 2D and 3D classification approaches in RELION were used to exclude data.
Replication	For cryoEM, two randomly divided half datasets were processed independently, and combined to give rise to the final structures. The resolution of the structure is assessed by comparing the two independent maps.<br>Over three independent measurements were made successfully for GTPase activity.
Randomization	Data collection for cryo-EM images are randomized by the data collection software. CryoEM particles were randomly divided into ODD and EVEN datasets, as standard approach implemented in RELION3.
Blinding	investigations are blinded to the data collection.

Reporting for specific materials, systems and methods

We require information from authors about some types of materials, experimental systems and methods used in many studies. Here, indicate whether each material, system or method listed is relevant to your study. If you are not sure if a list item applies to your research, read the appropriate section before selecting a response.

Materials & experimental systems

n/a	Involved in the study	
☐	☒	Antibodies
☐	☒	Eukaryotic cell lines
☒	☐	Palaeontology and archaeology
☒	☐	Animals and other organisms
☒	☐	Human research participants
☒	☐	Clinical data
☒	☐	Dual use research of concern

Methods

n/a	Involved in the study	
☒	☐	ChIP-seq
☒	☐	Flow cytometry
☒	☐	MRI-based neuroimaging

Antibodies

Antibodies used	ab14448, Abcam, is now available under product code ab13251.<br>Goat anti-mouse IgG 5nm gold conjugate, 0.5 ml, Ted Pella catalog number 17100-5. Dilution is 250 x
Validation	https://www.abcam.com/dynamin-1-antibody-d5-ab13251.html

Eukaryotic cell lines

Policy information about cell lines

Cell line source(s)	Expi293 cells were purchased from the American Type Culture Collection (cat. No. CRL-3216).
Authentication	None of the cell lines we used was authenticated by us.
Mycoplasma contamination	We did not test the cell lines for mycoplasma contamination.
Commonly misidentified lines (See ICLAC register)	none
